# BOHEMIA a cluster randomized trial to assess the impact of an endectocide-based one health approach to malaria in Mozambique: baseline demographics and key malaria indicators

**DOI:** 10.1186/s12936-023-04605-3

**Published:** 2023-06-04

**Authors:** Paula Ruiz-Castillo, Saimado Imputiua, Kexin Xie, Eldo Elobolobo, Patricia Nicolas, Julia Montaña, Edgar Jamisse, Humberto Munguambe, Felisbela Materrula, Aina Casellas, Xinwei Deng, Achla Marathe, Regina Rabinovich, Francisco Saute, Carlos Chaccour, Charfudin Sacoor

**Affiliations:** 1 ISGlobal, Hospital Clínic - Universitat de Barcelona, Barcelona, Spain; 2 Centro de Investigaçao em Saúde de Manhiça, Manhiça, Mozambique; 3 Department of Statistics, Virginia Tech, Blacksburg, VA USA; 4 Network Systems Science and Advanced Computing Division, Biocomplexity Institute, University of Virginia, Charlottesville, VA USA; 5 Department of Public Health Sciences, University of Virginia, Charlottesville, VA USA; 6 Harvard T.H. Chan School of Public Health, Boston, USA; 7 Ciberinfec, Madrid, Spain; 8 Universidad de Navarra, Pamplona, Spain

**Keywords:** Mozambique, Mopeia, BOHEMIA, Demographic survey, Mapping, Long-lasting insecticidal nets, Indoor residual spraying, Population structure, Malaria

## Abstract

**Background:**

Many geographical areas of sub-Saharan Africa, especially in rural settings, lack complete and up-to-date demographic data, posing a challenge for implementation and evaluation of public health interventions and carrying out large-scale health research. A demographic survey was completed in Mopeia district, located in the Zambezia province in Mozambique, to inform the Broad One Health Endectocide-based Malaria Intervention in Africa (BOHEMIA) cluster randomized clinical trial, which tested ivermectin mass drug administration to humans and/or livestock as a potential novel strategy to decrease malaria transmission.

**Methods:**

The demographic survey was a prospective descriptive study, which collected data of all the households in the district that accepted to participate. Households were mapped through geolocation and identified with a unique identification number. Basic demographic data of the household members was collected and each person received a permanent identification number for the study.

**Results:**

25,550 households were mapped and underwent the demographic survey, and 131,818 individuals were registered in the district*.* The average household size was 5 members and 76.9% of households identified a male household head. Housing conditions are often substandard with low access to improved water systems and electricity. The reported coverage of malaria interventions was 71.1% for indoor residual spraying and 54.1% for universal coverage of long-lasting insecticidal nets. The median age of the population was 15 years old. There were 910 deaths in the previous 12 months reported, and 43.9% were of children less than 5 years of age.

**Conclusions:**

The study showed that the district had good coverage of vector control tools against malaria but sub-optimal living conditions and poor access to basic services. The majority of households are led by males and Mopeia Sede/Cuacua is the most populated locality in the district. The population of Mopeia is young (< 15 years) and there is a high childhood mortality. The results of this survey were crucial as they provided the household and population profiles and allowed the design and implementation of the cluster randomized clinical trial.

*Trial registration* NCT04966702.

## Background

After years of progress, the estimated number of malaria cases and deaths have increased since 2015 [1]. Moreover, disruptions of the health system caused by the COVID-19 pandemic have worsened the negative trends. According to the World Malaria Report of 2021, 95% of malaria cases and 96% of malaria deaths occur in the World Health Organization (WHO) Africa region. Mozambique accounts for 3.8% of global malaria deaths, being one of the 11 countries that are part of the High Burden High Impact strategy set by the WHO Global Malaria Programme in 2019 to tackle malaria in the most affected areas [2].

Mozambique has a very heterogeneous malaria transmission, with areas of very high prevalence in the North, up to 57% in Cabo Delgado and 44% in Zambezia in children 6–59 months, but significantly lower in the South, going down to 1% in Maputo province [3]. Given the high levels of transmission if the northern areas, where traditional vector control tools, such as indoor residual spraying (IRS) and long-lasting insecticidal nets (LLINs) have been in place for years, novel strategies to decrease the malaria burden are urgently needed.

The district of Mopeia, in Zambezia was chosen to carry out a Unitaid-funded cluster randomized trial (cRCT) to test an innovative tool against malaria. The Broad One Health Endectocide-based Malaria Intervention in Africa (BOHEMIA) trial evaluated the mass drug administration (MDA) of ivermectin to humans only and humans and livestock simultaneously to decrease malaria transmission by killing the mosquitoes that bite on treated blood sources [4]. The project was implemented by the Centro de Investigação em Saúde de Manhiça (CISM), which established in Mopeia a research site in 2016 given the high burden of malaria in the district.

In many low- and middle-income countries, including Mozambique, demographic data are often incomplete, riddled with errors, rough estimates and interpolations, or simply out of date. The lack of high-quality demographic data poses a challenge to health research, as incorrect estimates of values such as “population at risk” can lead to downstream biases in epidemiological studies as well as funding priorities. Comprehensive demographic enumeration has been established in many places, creating geographic areas where data are timely and of high quality, such as the Health and Demographic Surveillance Systems (HDSS) [5, 6], as implemented by CISM in the South of Mozambique [7]. A demographic surveillance platform was established in 1997 in Manhiça and has been updated throughout the years [8, 9]. However, developing a HDSS is complex and time consuming. Alternatively, simplified demographic platforms that collect household and health data can be established in preparation for a research study, such as the one in Magude, which served as the groundwork for a large-scale MDA malaria elimination strategy [10]. Prior to BOHEMIA, a demographic survey was carried out in Mopeia in 2016 in preparation for the COST study (Cost-effectiveness evaluation of vector control strategies in Mozambique), which provided knowledge of the area (i.e. geographical, demographic, and information on health system usage [11]. Due to changes in the local administrative units and constant population movements in Mopeia, updated demographic data was required in preparation for the BOHEMIA MDA.

The main objective of the BOHEMIA demographic survey was to map all households in the district in order to create the study clusters, which would then be randomized to the different arms of the cRCT. The survey collected information about the number and exact location of households as well as the total population in the Mopeia district. Additionally, the output of this survey served to design the implementation plan for all BOHEMIA work packages. Lastly, this district-wide demographic survey was the first project activity, which enabled the establishment of contact with the community and engagement with them before the cRCT.

Here, the results of the BOHEMIA demographic survey, a prospective, descriptive study, that mapped the households in the Mopeia district and captured population characteristics, as well as all-cause mortality and coverage of key vector control interventions, are presented.

## Methods

### Study area

**Fig. 1:**
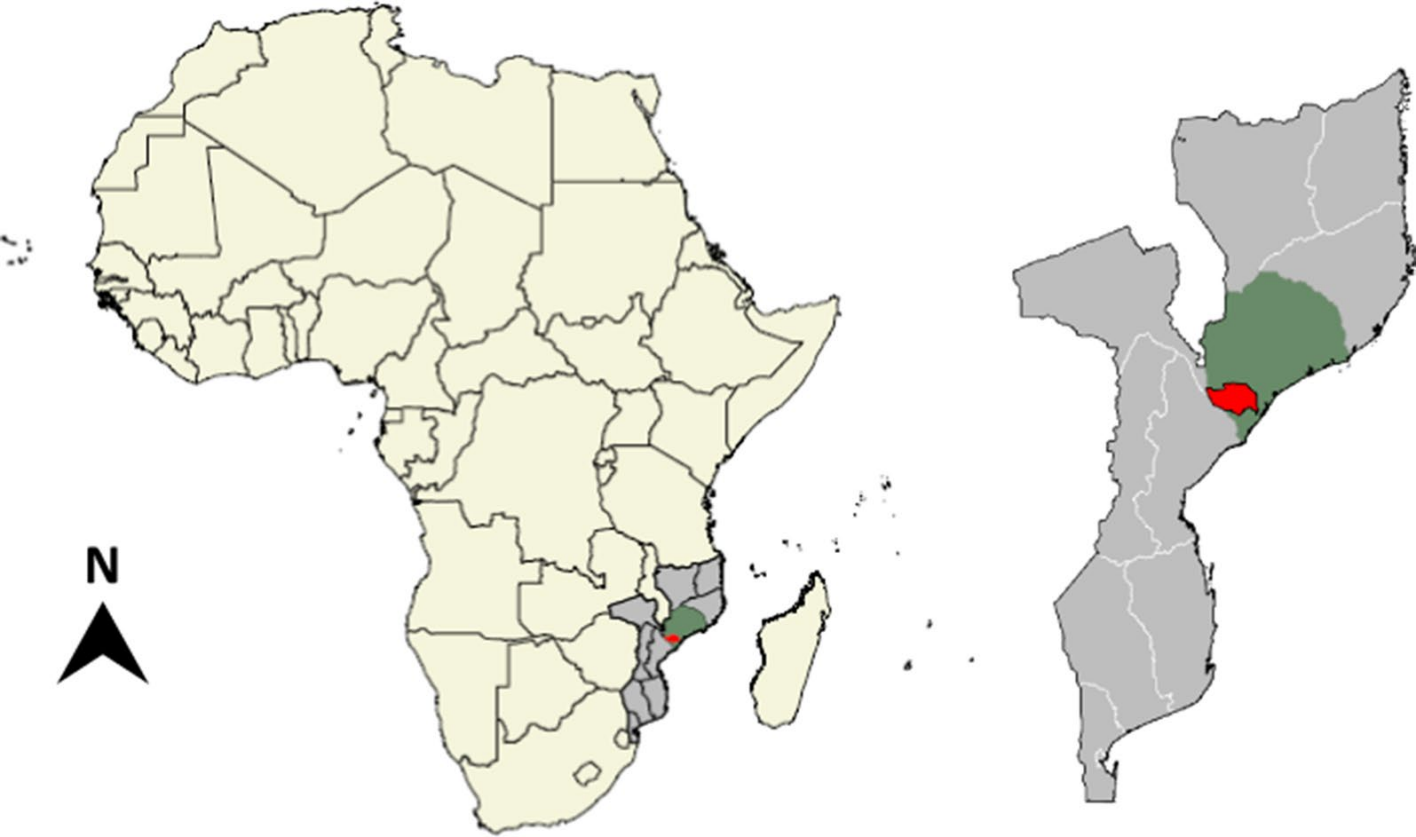
**a** Map of Mozambique (grey), Zambezia province (green), and Mopeia district (red); **b** Enlarged map of Mozambique, Zambezia, and Mopeia

Mopeia district has an area of 7,671 km^2^ and is located in the southwest of the Zambezia province in the Centre of Mozambique. Zambezia is the second most populous province of Mozambique (5,854,843 inhabitants in 2017) with a total surface area of 103,478 km^2^ [12] and a total of 238 health units. The district of Mopeia is bordered by the Morrumbala district to the North, Luabo district and Sofala province to the South, Nicoadala and Inhassunge districts to the East, and Sofala and Tete provinces to the West (Fig. 1). Mopeia is divided in two areas, the highlands in the North and the flood plains of the Zambezi River in the South. Mopeia is a relatively isolated district with harsh climatic conditions that include changing rainy seasons and cyclones. There are two distinct seasons, a wet season, which usually lasts from November to April, and a dry one from May to October.

The total population of Mopeia was estimated by the Instituto Nacional de Estatística (INE) to be 136,520 people in 2017 [13]. The district is distributed in 2 administrative posts (Mopeia and Campo), 8 localities (Mopeia Sede/Cuacua, Sambalendo/Chimuara, Nzanza, Rovuma/Conho, Campo Sede, Catale, Luala, Mungane), a number of villages and, at the smallest administrative level, several hundred hamlets (*bairros* in Portuguese), which present some degree of variation over time. The organizational structure consists of a district governor, chiefs of the administrative posts and localities, and at a smaller level, hamlet leaders, and *muenes* who are community leaders.

**Fig. 2:**
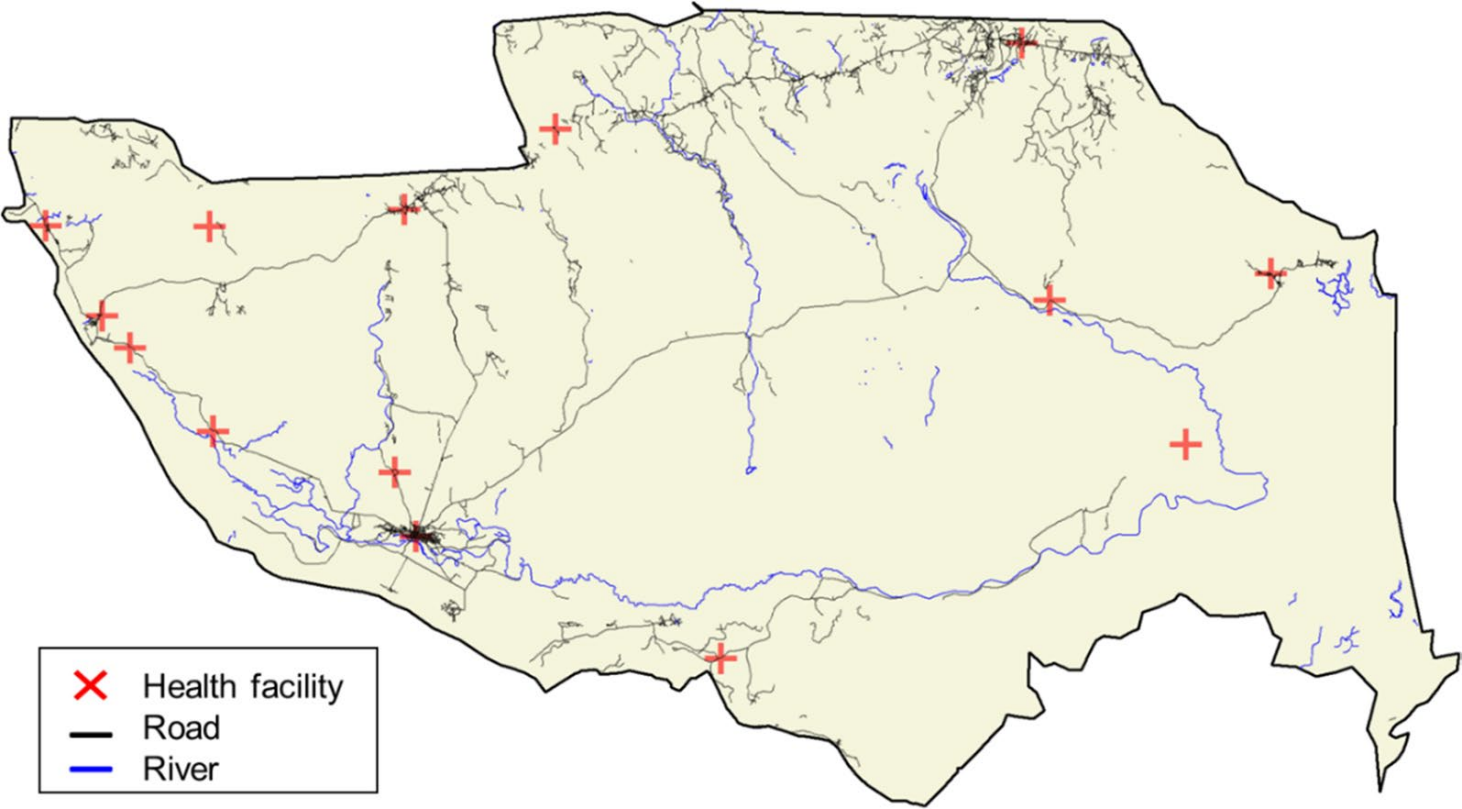
Map of Mopeia district

The district of Mopeia has a road network of approximately 700 km (Fig. 2). There is one main road that crosses the Northern part of the district but most of the roads are tertiary and dirt roads in poor state during the rainy season. There is 3G mobile internet connectivity only in some areas. The district has 13 health posts with no inpatient capacity, plus one health facility that serves as the district hospital, located in Mopeia Sede/Cuacua (Fig. 2). In 2020 there were over 224,000 outpatients seen in the health facilities and 407 inpatients attended to the district hospital. There is also a platform of approximately 55 community health workers (*agentes polivalentes elementares*, APEs from the acronym in Portuguese), who serve as the link between the health system and the remote communities by working on health promotion, disease prevention (e.g. deworming, providing vitamins), and treatment (e.g. malaria, pneumonia and diarrhoea).

Similar to other rural areas of Mozambique, Zambezia has a high burden of communicable diseases, with a HIV prevalence of 12.6% among those aged 15–49 years [14] and an estimated incidence of tuberculosis of 900 cases per 100,000 people in Mopeia. In addition, the area is prone to diarrheal diseases and cholera outbreaks. Mopeia is a district representative of the North of Mozambique, with a very high malaria burden. A recent study in the area detected an incidence of 4.5 cases per child-year at risk and 62–75% prevalence in under five years old at the peak of the rainy season [15]. The primary vectors in Mopeia are *Anopheles funestus* sensu lato (*s.l*.) and *Anopheles gambiae* *s.l.* [16]. Interventions for malaria in Mopeia are centered on vector control with IRS and LLINs [17], as well as case management and intermittent preventive treatment of pregnancy (IPTp).

### Study design and definitions

This was a prospective descriptive study in which data were collected at the household level and consisted of 4 phases: (a) reconnaissance, (b) community engagement, (c) enumeration, (d) household data collection.

- *Reconnaissance* was designed to map all the administrative units of the district, down to the level of village and hamlet, as well as to create 3-letter codes to identify hamlets. Even though the district administrative posts and wards were known, the names and borders of villages and hamlets had varied from those previously recorded in 2016 [15]. Reconnaissance consisted first on a set of phone calls to the high-level leaders to inquire about hamlet leaders and their contact information; and second, in person meetings with the hamlet leaders to collect data about the approximate number of households, closest health facility, 3G connectivity, and accessibility, among others. This phase took place between January and September of 2020, with interruptions due to the COVID-19 pandemic. It was carried out by a team of CISM staff plus 5 field workers.
- *Community engagement* consisted of a series of meetings with the key political, administrative, and health authorities, traditional leaders as well as with community leaders. The community mobilizers presented the BOHEMIA study and the objectives of the demographic survey. They explained the data collection process, timelines, and inclusion criteria. The team asked the local leaders to collaborate by disseminating the information to the community and guiding the field workers around the hamlet if necessary.
- *Enumeration* consisted of the identification of all households in Mopeia through a household identification number (HouseholdID) and the capture of household location GPS coordinates. The enumeration team were given pre-generated HouseholdIDs for each hamlet, using the information collected during the reconnaissance. HouseholdIDs consisted of a 6-digit alphanumeric code where the first three characters were the 3-letter codes of each hamlet and the last three were ascending numbers for each household (e.g. ABC-001, ABC-002 …). HouseholdIDs were painted in black or white on the front wall of the households after verbal consent. This phase was carried out by a team of 20 field workers.
- *Household data collection* consisted of the data collection phase through the demographic survey. Upon inform consent, participants were asked to list and provide basic information of all household members, as well as to identify the household head. All individuals were assigned a personal identification number (ExtID) based on the corresponding HouseholdID (e.g. ABC-001-001, ABC-001-002). Data on household characteristics, livestock ownership, malaria prevention tools, and all-cause mortality was also collected. The demographic survey was done by a team of 100 field workers and 12 supervisors.

Households that did not participate in the study either because they refused or there was nobody present or able to respond after two attempts were only geolocated. This allowed to identify areas with high refusal levels and reinforce community engagement, as well as to account for all households in the district.

### Data collection tools and data management

Data was collected by field workers through digital forms using Open Data Kit (ODK, https://opendatakit.org) in Android tablets. The data from areas with 3G connectivity was sent to the CISM local server daily; for those without 3G connectivity, a data upload plan was developed in which field supervisors would take the tablets to 3G areas frequently. A system was developed by the project data manager to review the data soon after it arrived at the local server. The system was programmed to automatically detect and notify the local data management team of anomalous or erroneous data, who then investigated each data entry incident. After investigation, the local team sent the data manager a correction of the erroneous data or confirmed that the data was correct and no changes were needed. In parallel, the site data manager sent correction submissions of anomalies detected in the field. An audit trail of all data modifications was generated automatically and copies of the raw uncorrected data were stored throughout the project.

### Data analysis

All descriptive analyses presented here were conducted using R 4.1.2 [18]. Calculations were made to determine the median and interquartile range (IQR, the first and third quantiles) or mean and standard deviation, for the continuous variables without considering missing values. The frequency and the percentage distribution of each category were computed for the categorical variables with the missing value in the denominator.

## Results

### Mopeia geographical hierarchy

A total of 40 villages and 358 hamlets were mapped and listed during Reconnaissance.

### Household information

Community engagement, enumeration and household data collection took place from October to March 2021. A total of 27,928 households were enumerated and 25,550 households participated in the survey whereas 546 households either refused to participate or were absent twice. Due to accessibility issues during the rains 1832 households were enumerated but not visited for the demographic survey.

### Household size and socio-economic characteristics

The average household size in Mopeia was of 5 inhabitants, with 1,170 households inhabited by only one person (Table 1). The majority of the households identified a household head, who was male (76.9%), with a median age of 38 years.

**Table 1:** Household characteristics and conditions in Mopeia

Household characteristic (N = 25,550)		Frequency	Percentage (%)
Household size^a^		5	[3, 7]
Single resident households		1170	4.6
Household head			
Sex	Male	19,638	76.9
	Female	5912	23.1
Age^a^		38	[28,51]
Household constructions (i.e.; built structures)	1	16,031	62.7
	2	6058	23.7
	3 or more	3451	13.5
	Unknown	10	0.04
Household constructions with sleeping rooms	1	18,275	71.5
	2	4987	19.5
	3 or more	2211	8.6
	Unknown	77	0.30
Household type	Traditional mud house	9430	36.9
	Hut	7411	29.0
	Precarious	4990	19.5
	Conventional house	3364	13.2
	Other	331	1.3
	Unknown	24	0.1
Household wall material	Adobe	13,938	54.6
*(households could report more than one material)*	Bamboo	6647	26.0
	Brick block	4004	15.7
	Wood	2496	9.8
	Palm tree	2397	9.4
	Tin	1210	4.7
	Tinned wood	1183	4.6
	Bark	1065	4.2
	Other	8670	33.9
Main water source for cooking and hygiene	Hole protected with hand pump outside	12,922	50.6
	Unprotected well outside	4232	16.6
	Fountain	2743	10.7
	Water from river	2081	8.1
	Protected well outside	1228	4.8
	Lagoon	586	2.3
	Other	1715	6.7
	Unknown	43	0.2
Time to water source	Under 10 min	8135	31.8
	Between 10–30 min	11,658	45.6
	Between 30–60 min	4534	17.7
	More than 60 min	1216	4.8
	Unknown	7	0.03
Main source of energy for lightning	Batteries	17,577	68.8
	Electricity	2976	11.7
	Firewood	3057	12.0
	Solar panel	1191	4.7
	Other	646	2.5
	Unknown	103	0.4
Ownership of goods	Cell phone	9829	38.5
*(households could report more than one item)*	Radio	6301	24.7
	TV	1909	7.5
Livestock ownership	No livestock	23,539	92.1
	Pigs	1881	7.4
	Cattle	86	0.3
	Pigs and cattle	39	0.2
	Unknown	5	0.02
Average number of pigs per household^b^		4.7	6.2
Average number of cattle per household^b^		4.9	5.2

^a^Median [Interquartile range]

^b^Mean (standard deviation)

Most of the constructions are traditional mud houses (36.9%) or huts (29.0%), and are made of materials, such as adobe (54.6%) and bamboo (26%). The study found a proportion of households, which even reported card board and plastic bags as the main materials for the house walls (under category “Other” in Table 1).

Half of the households in Mopeia had access to water through a protected hole with a hand pump outside (50.6%), whereas 16.6% of the houses had an unprotected well outside. Several households did not have direct access to water and they took it from a shared fountain (10.7%) or even directly from the river (8.1%) and a lagoon (2.3%). The majority of them were close to their source of water. Most households reported using batteries (68.8%) for lighting whereas only 11.7% used electricity, and 12.0% firewood. The majority of households did not own cattle or pigs, a total of 7.4% of households owned pigs in Mopeia, and a few (0.3%) had cattle. Out of those that had livestock, the average number of pigs per household was 4.7 and of cattle 4.9.

### Malaria prevention

The vast majority of households in the district owned at least one LLIN (90.7%) and 54.1% of households met the definition of universal coverage of having at least one LLIN per every two people (Table 2). The majority of households had obtained at least one LLIN in the past year (85.5%). A 71.1% of the households in the district received IRS in the last 12 months as part of a USAID-PMI campaign.

**Table 2:** Coverage of malaria prevention tools in Mopeia

Households (N = 25550)	Frequency	Percentage (%)
IRS		
Households that received IRS in the past 12 months	18165	71.1
LLIN ownership		
Number of LLINs per household		
0	2364	9.3
1	5676	22.2
2	7723	30.2
3	6751	26.4
> 3 nets	3036	11.9
Households with universal LLINs coverage (1 net/2 people)	12549	54.1
Households that obtained the nets *(households could report more than one LLIN)*		
1 year ago or less	21846	85.5
Within the past 1–3 years	2500	9.8
More than 3 years	352	1.3

Individuals (N = 131818)	Frequency	Percentage (%)
Individuals living in households with universal coverage	51354	38.9

### Individual information

**Fig. 3:**
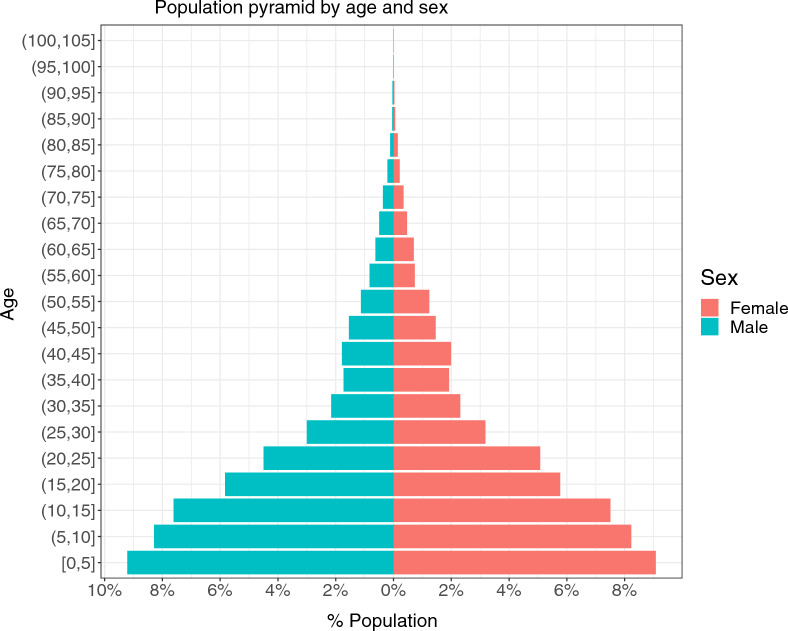
Population pyramid by age and sex

The survey registered a total of 131,818 inhabitants of which 66,549 (50.6%) were women (Fig. 3 and Table 3). The age structure of the population in Mopeia was 18.3% under 5 years of age, 31.6% between 5 and 15 years old, 47.4% people of working age between 15 and 64 and 2.7% over 64 years old, with a median age of 15 years. The population density varied by locality, with the largest group living in the district capital Mopeia Sede/Cuacua (31.7%), Sambalendo/Chimuara (22.1%), and 13.8% in Campo Sede.

**Table 3:** Population structure

Individuals (N = 131818)		Frequency	Percentage (%)
Age^a^ (in years)		15	[6,27]
Age group (in years)	(0, 5)	24111	18.3
	[5, 15)	41702	31.6
	[15–64)	62443	47.4
	≥ 64	3562	2.7
Sex	Male	65269	49.5
	Female	66549	50.6
Individuals per locality			
	Mungane	3200	2.4
	Catale	5290	4.0
	Rovuma/Conho	8042	6.1
	Nzanza	10433	7.9
	Luala	15676	11.9
	Campo sede	18165	13.8
	Sambalendo/Chimuara	29189	22.1
	Mopeia sede/Cuacua	41823	31.7

^a^Median [Interquartile range]

### Mortality in the past 12 months

**Fig. 4:**
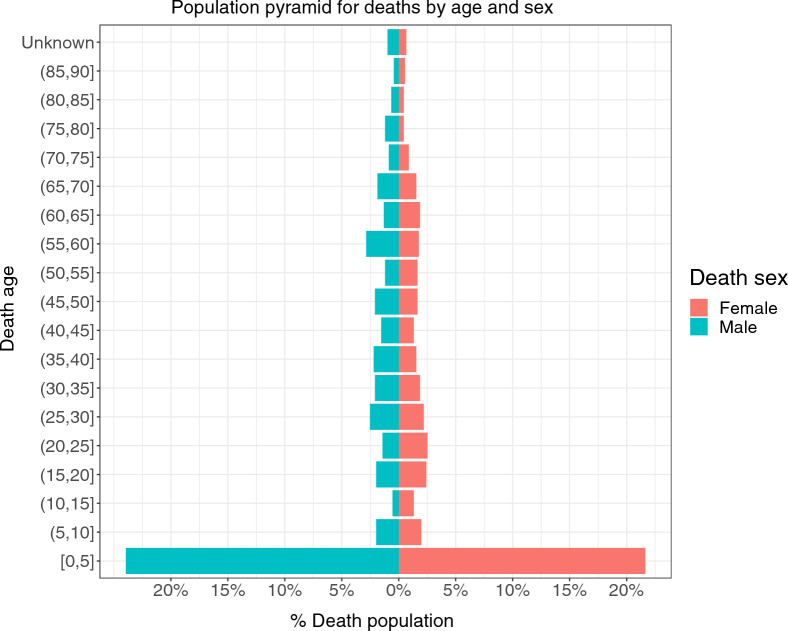
Population pyramid for deaths occurred in the last 12 months by age and sex

A total of 859 households (3.4%) in Mopeia reported the death of a household member in the prior 12 months; of these, 5.1% of households reported more than one death (Table 4). Regarding the age distribution, the under 5 years old group was disproportionally represented with almost half of the deaths being from that age range (43.9%) (Table 4 and Fig. 4). Moreover, out of the total number of deaths, 24.6% occurred in the first year of life. In terms of the location of the deaths, most occurred at home (80.7%) and only 15.8% at the hospital. Similar age and death location distribution were observed by sex.

**Table 4:** All-cause mortality in the past 12 months in Mopeia at the household and individual level

Deaths in the past 12 months (N = 25550)	Frequency	Percentage (%)		
Households that reported death(s) in the last 12 months	859	3.4		
Households with 1 death^a^	815	94.9		
Households with > 1 death^a^	44	5.1		

Dead subjects’ characteristics (N = 910)	Female (N = 439)		Male (N = 471)	
	Frequency	Percentage (%)	Frequency	Percentage (%)
Deaths by age (in years)				
(0, 1)	100	22.8	124	26.3
[1–5)	92	20.9	84	17.8
[5–15)	33	7.5	32	6.8
[15, 64)	168	38.3	170	36.1
≥ 64	40	9.1	52	11.0
Unknown	6	1.4	9	1.9
Death location				
At home	360	83.0	374	79.4
In the hospital	63	14.3	76	16.1
On the way to the hospital	8	1.8	6	1.3
Accident location	1	0.2	3	0.6
Traditional healer’s home	1	0.2	3	0.6
In the church	2	0.5	–	–
Other	4	0.9	9	1.9

^a^N = 859, number of households that reported death(s) in the last 12 months

## Discussion

A district-wide demographic survey was conducted in Mopeia in order to map, identify, and characterize the households in the area, as well as to determine the population size and structure. The key output of the demographic survey was the creation of the study clusters, which were built based on the population of children under five years of age. In addition, the demographic survey was crucial to planning the implementation of all field activities related to the clinical trial and the other BOHEMIA work packages (including drug amount estimation, number of field personnel). These data served to draw the randomization and sampling schemes of the cRCT, as well as to identify relevant information for the study (e.g. livestock ownership or lack thereof). Lastly, mapping all households in the district allowed the calculation of the proportion of households reached by the cRCT.

The reconnaissance activity was a key starting point when working in such a remote place as Mopeia. It served to further know the size of the district, the accessibility, and the 3G in the district hamlets, which allowed for effective planning of all other field activities. A total of 27,928 households were enumerated in the study (including those that did not participate). The majority of the district households (25,550, 91.5%) were mapped through their GPS coordinates, identified with a household ID, and a household member responded the demographic survey. A total of 546 households (2%) declined to participate in the study or had nobody present after two attempts, which indicates the success of the community engagement and demonstrates the importance of such activities in studies of this nature.

The results of the demographic survey indicate that the living conditions in Mopeia are sub-standard and often not adequate for the intense rains and cyclones that affect the district. Access to electricity is low. According to the National Malaria Survey (Inquérito Nacional sobre Indicadores de Malária em Moçambique 2018, IMM), only 13.3% of households in Zambezia have electricity, which is close to the 11.7% identified in the study. In-country definitions for improved water sources include those that are physically protected from external contamination such as piped water, fountains, protected wells, protected holes with manual pumps, rain water, bottled water, and water from a tank truck [3]. Any water sourced from unprotected wells, or surface water (e.g. lake, river, lagoon) is considered unimproved. Based on these indicators, the IMM reports that 54.4% of households in Zambezia have access to improved water systems, whereas the study identified 66.1% for Mopeia (considering “Unknown” and “Other” as unimproved sources). According to the WHO/UNICEF monitoring programme [19], access to basic drinking water means obtaining water from an improved source taking less than 30 min total to fetch it. A total of 45.6% of the households in Mopeia reported having access to water between 10 and 30 min and 31.8% under 10, but a 17.7% reported taking between 30 and 60 min, and 4.8% reported taking more than one hour. These results show that not all households in Mopeia have access to basic water services, which can lead to poor hygiene conditions and the spread of infectious diseases.

In rural areas of low-income countries, owning livestock may represent a significant percentage of household economy, since the animals can be sold or used for self-consumption or physical work [20]. However, only 7.9% of households in Mopeia reported owning pigs and/or cattle (no other livestock was asked about). Out of the households owning pigs and/or cattle, most owned pigs, with very few households owning cattle. This might be indicative of the poverty rates in Mopeia or that families own other livestock, such as goats, chickens.

Mopeia is an area of high malaria burden, as a recent study indicated 62–75% prevalence in children less than five years at the peak of the rainy season [15]. However, the district has great access, coverage, and renewal of LLINS as shown by the demographic survey. 90.7% of the households in Mopeia district had at least one LLIN, which is higher than what the IMM reported for Zambezia province in 2018, 81%, and 54.1% of the households in Mopeia have universal coverage of LLINs, defined as one LLIN per two household members. The IRS coverage reported in 2018 for Zambezia was 23,5% and the study registered 71.1% in Mopeia in 2020. Having such a good coverage vector control tools and still, high malaria burden made Mopeia a suitable district to evaluate a complementary vector control strategy.

In terms of population, 131,818 people were registered, which is less than the INE estimations but the study missed those living in households that refused or for which the household members were absent twice. The population structure in Mopeia is typical for a sub-Saharan country, in which the largest age group is the youngest children and the shape of the pyramid narrows as the population age increases. There are slightly more women than men and there are only 23.1%. female household heads, which shows that the home-based structures in Mopeia are mostly male dominated.

The death age structure in Mopeia is completely skewed towards the young population. Out of the total deaths reported in the prior 12 months, the largest number occurred in children under 5 years old (43.6%). This is aligned with the results of the 2019 Countrywide Mortality Surveillance for Action (COMSA), which reported that 37% of deaths in the Zambezia province were of children under five years old [21]. Moreover, most of the deaths reported in Mopeia occurred during the first year of life. This demographic survey did not inquire about the cause of death, but based on previous reports from the national survey of cause of death (Inquerito Nacional sobre Causas de Mortalidade), malaria is the major cause of deaths of children under five years old in Zambezia, with almost 40% of deaths attributable to this disease [22]. Other major causes of death reported among children in Zambezia are HIV, pneumonia, and diarrhoeal diseases, showing a high burden of communicable diseases. Lastly, the majority of deaths occurred at home, which might result from poor access to health care and under resourced health services. Although not asked in the demographic survey, it might also result from poor health-seeking behaviour. A key consequence of this was the need for reinforced active surveillance of severe adverse events and adverse events at community level during the cRCT.

The main limitation of the study is the size and depth of the survey, since it did not allow for detailed individual data collection. All the information was collected at the household level, without any person level data besides basic demographic data of the household members. Another limitation is that, the study did not use satellite imagery in order to map and identify the area of work prior to field operations, which has been previously used in similar interventions such as IRS campaigns [23, 24]. Although this strategy has shown to be quick and cheap, and has helped to ensure all households in an area are included, this demographic survey was carried out in a very rural, disperse, and remote area with dense vegetation, making very challenging to differentiate among the different types of constructions and distinguish them among the vegetation. Other limitations of the study arise from the difficulty of working in a remote area such as Mopeia. The team faced accessibility issues which significantly slowed down field activities and several households were found destroyed or abandoned due to the harsh weather conditions. In addition, data collection in areas without 3G connectivity represented a great operational challenge. Overall, this experience highlighted the difficulties to be encountered at the time of the MDA or any community-based intervention that requires a large-scale field deployment.

## Conclusions

The demographic survey in Mopeia was the first step in preparation for the deployment of a cRCT to test the use of ivermectin at community level to reduce malaria transmission. In contrast to a programmatic health intervention, a controlled study like BOHEMIA required a comprehensive mapping of the area of work prior to implementation. The data of the demographic survey served to build the study clusters and tox plan the deployment of the large-scale MDA.

Despite the great coverage of vector control tools in Mopeia, the local malaria burden is high and the living conditions are sub-optimal, with a number of households not having access to basic services such as electricity and/or improved water sources. The demographic survey showed that population in Mopeia is young, with a median age of 15 years old. The majority of deaths are concentrated in children and occur at home, indicating a poor access to health care and the need to design strategies to decrease disease and mortality in the area.

## Data Availability

The datasets generated and/or analysed during the current study are available in the BOHEMIA repository, [https://dataverse.csuc.cat/dataverse/bohemia].

## Abbreviations

BOHEMIA: Broad one health endectocide-based malaria intervention in Africa
CISM: Centro de Investigação em Saúde de Manhiça
COST: Cost-effectiveness evaluation of vector control strategies in Mozambique
cRCT: Cluster randomized controlled trial
HDSS: Health and demographic surveillance system
HouseholdID: Household identification number
INE: Instituto Nacional de Estatística
IPTp: Intermittent preventive treatment
IRS: Indoor residual spraying
LLINs: Long-lasting insecticide-treated nets
MDA: Mass drug administration
ODK: Open Data Kit
